# Interactions Between *Clostridioides difficile* and Fecal Microbiota in *in Vitro* Batch Model: Growth, Sporulation, and Microbiota Changes

**DOI:** 10.3389/fmicb.2018.01633

**Published:** 2018-07-24

**Authors:** Sabina Horvat, Maja Rupnik

**Affiliations:** ^1^Department of Microbiology, Faculty of Medicine, University of Maribor, Maribor, Slovenia; ^2^Centre for Medical Microbiology, National Laboratory for Health, Environment and Food, Maribor, Slovenia

**Keywords:** *Clostridioides* (*Clostridium*) *difficile*, gut microbiota, sporulation, colonization, pathogenesis, CDI

## Abstract

Disturbance in gut microbiota is crucial for the development of *Clostridioides difficile* infection (CDI). Different mechanisms through which gut microbiota influences *C. difficile* colonization are known. However, *C. difficile* could also affect gut microbiota balance as previously demonstrated by cultivation of fecal microbiota in *C. difficile* conditioned medium. In current study, the interactions of *C. difficile* cells with gut microbiota were addressed. Three different strains (ribotypes 027, 014/020, and 010) were co-cultivated with two types of fecal microbiota (healthy and dysbiotic) using *in vitro* batch model. While all strains showed higher sporulation frequency in the presence of dysbiotic fecal microbiota, the growth was strain dependent. *C. difficile* either proliferated to comparable levels in the presence of dysbiotic and healthy fecal microbiota or grew better in co-culture with dysbiotic microbiota. In co-cultures with any *C. difficile* strain fecal microbiota showed decreased richness and diversity. Dysbiotic fecal microbiota was more affected after co-culture with *C. difficile* than healthy microbiota. Altogether, 62 OTUs were significantly changed in co-cultures of dysbiotic microbiota/*C. difficile* and 45 OTUs in co-cultures of healthy microbiota/*C. difficile*. However, the majority of significantly changed OTUs in both types of microbiota belonged to the phylum *Firmicutes* with *Lachnospiraceae* and *Ruminococcaceae* origin.

## Introduction

*Clostridium difficile*, recently reclassified as *Clostridioides difficile* (Lawson et al., 2016), is an ubiquitous, anaerobic, spore-forming, Gram-positive bacterium that is currently the leading cause of nosocomial and community associated diarrhea worldwide (Martin et al., 2016). *C. difficile* infection (CDI) outcome can range from asymptomatic colonization to diarrhea and more severe, life-threatening pseudomembranous colitis (Rupnik et al., 2009; Leffler and Lamont, 2015). CDI is a toxin mediated disease. Two toxins, A and B, are well established as virulence factors, while the presence of the third toxin, binary toxin CDT, is less clear but seems to contribute to disease severity (Gerding et al., 2014).

Success of enteric pathogen to cause a disease depends mainly on two phases, i.e., the initial colonization phase of pathogen in the gut, followed by pathogen-promoting physiological changes in the gut important for its maintenance (Ferreyra et al., 2014). The colonization of *C. difficile* is strongly associated with disturbance in gut microbiota (i.e., dysbiosis) and several studies explored the mechanisms through which gut microbiota influences *C. difficile* (Britton and Young, 2012; Pérez-Cobas et al., 2015). While for some enteric pathogens (e.g., *Salmonella*) mechanisms involved in the pathogen-promoting physiological changes in the gut have already been described (Stecher et al., 2007), little is known about possible impact of *C. difficile* on gut microbiota. In our previous work, we have shown that *C. difficile* conditioned medium has specific effect on fecal microbiota and that nutrient competition is the most likely mechanism (Horvat et al., 2017). Additionally, changed bacterial groups detected in *in vitro* model using *C. difficile* conditioned medium were comparable to those described in CDI patients.

In this study, we have investigated the interactions between *C. difficile* vegetative cells and fecal bacterial communities in a simple *in vitro* model. Several lines of evidence supports the need for dysbiotic gut microbiota for successful *C. difficile* colonization, such as low carriage rates in healthy population, need of antibiotic pretreatment of animals in animal models of CDI and association of dysbiotic gut microbiota with CDI in humans. However, recent increase in community cases without previous antibiotic exposure (Gupta and Khanna, 2014) suggests that *C. difficile* can colonize gut also in the presence of non-dysbiotic microbiota. In line with this observations are *in vivo* and *in vitro* studies demonstrating capability of *C. difficile* to colonize the gut even in the presence of complex microbiota (Robinson et al., 2014) and showing that in the absence of some protective species (i.e., *C. scindens*) colonization with *C. difficile* even without antibiotic treatment can result in a significant compositional perturbation of gut microbiota in animal models (Studer et al., 2016). We have therefore compared two types of fecal samples, one representing healthy microbiota and the other representing dysbiotic microbiota. Healthy microbiota was obtained from healthy donors. As a proxy for dysbiotic microbiota fecal samples sent to routine diagnostic laboratory for testing for *C. difficile* were used. Such samples were shown before to clearly group separately from healthy volunteer samples (Skraban et al., 2013; Horvat et al., 2017). The interactions of both fecal microbiota types were tested with three different *C. difficile* strains as representatives of globally widespread PCR ribotypes (027, 014/020, and 010) and three different toxin production types (A+B+CDT+, A+B+CDT-, A-B-CDT-). The strains were selected with the objective to cover large diversity within *C. difficile* species. The aim was not to compare ribotype or toxinotype association with changes in interactions with gut microbiota as only a single representative was used.

## Materials and Methods

All described cultivation procedures were performed anaerobically at 37°C in an anaerobic workstation (10% CO_2_, 10% H_2_, and 80% N_2_) (Don Whitley Scientific).

### *C. difficile* Strain Selection and Preparation of Vegetative Cells

Three different *C. difficile* strains, belonging to PCR ribotypes 010, 014/020, and 027, were selected from our *C. difficile* strain collection (strain designations ZZV15-6684, ZZV11-3236, ZZV09-2033). Selected strains originated from humans and were isolated between the years 2009 and 2015. Strains were plated onto Columbia agar with 5% horse blood (BioMerieux) and incubated for 48 h. Single colony of each strain was transferred into 5 ml of pre-reduced Wilkins Chalgren Anaerobe Broth (WCAB) (Oxoid) and incubated for 17 h. Overnight cultures of each strain were once again transferred to freshly prepared and pre-reduced WCAB growth medium (1:500, v:v) and incubated for 17 h to obtain suspensions of *C. difficile* vegetative cells with up to 2 × 10^8^ colony forming units (CFUs) per ml, respectively.

### Fecal Samples Collection and Preparation of Fecal Inocula

*C. difficile* negative fecal samples were randomly selected from (i) non-diarrheal samples sent for *C. difficile* testing (male: *n* = 5, female: *n* = 5, all aged under 65 years) (dysbiotic microbiota) and (ii) samples of healthy individuals (male: *n* = 3, female: *n* = 2, all aged under 65 years) (healthy microbiota). Healthy individuals had not taken antibiotics for at least 3 months prior to donation. Healthy individuals have given informed consent and agreed to use the samples for the experiments. The use for laboratory samples without informed consent was approved by National Medical Ethic Committee.

After donation samples were stored at 4°C for maximally 24 h. Aliquots of 0.2 g of each specimen were prepared and frozen at -80°C until further processing. Based on our previous comparisons of individual and pooled samples and published studies (Aguirre et al., 2014) we used pooled samples for experiments with fecal microbiota. For pooled slurry preparation each specimen was thawed and diluted in pre-reduced WCAB to obtain 10% fecal slurry. Equal amounts of fecal slurries from each group (i.e., dysbiotic and healthy) were pooled, added to pre-reduced WCAB growth medium (1:100, v:v) and incubated overnight to prepare fecal inocula for *in vitro* cultivation.

### *In Vitro* Model Set Up of *C. difficile* and Fecal Microbiota Co-cultivation

Overnight culture of fecal inoculum (50 μl) was combined with suspensions of *C. difficile* vegetative cells (50 μl) in pre-reduced WCAB growth medium (4.9 ml) in a 6-well plate (Sarstedt). Initial concentration of *C. difficile* in co-cultures was approximately 2 × 10^6^ CFU/ml. Cultures were gently mixed and incubated for 3 days. Monocultures of fecal microbiota or *C. difficile* grown in freshly prepared and pre-reduced WCAB growth medium were used as controls. Each described growth condition was done in triplicate. Samples (5 ml) were taken after 72 h incubation period and screened for total viable cell count and spore count of *C. difficile* (described below). After centrifugation at 10,000 rpm for 10 min, pellets were used for total bacterial DNA extraction. Additionally, fecal pooled sample before any cultivation (i.e., fecal input sample) was included as a control for 16S rDNA sequencing to test for changes of microbiota due to *in vitro* cultivation.

### *C. difficile* Growth and Sporulation

For *C. difficile* total viable cell count serial dilutions were prepared in pre-reduced 0.9% sterile saline and plated onto pre-reduced chromID *C. difficile* agar plates (BioMerieux). *C. difficile* spores were defined as CFUs on chromID *C. difficile* agar plates after treatment with 100% ethanol for 30 min. The frequency of sporulation was determined as the percentage of ethanol resistant cell count relative to total cell count. One-way ANOVA was used in the statistical analysis with Bonferroni correction to determine statistically significantly different samples.

### Isolation of the Total Bacterial DNA and 16S rDNA Amplicon Sequencing of Microbiota

The extraction of bacterial DNA was performed with the QIAamp Fast Stool DNA Mini Kit (Qiagen) after the mechanical disruption (speed 7,000 for 70 s) with the SeptiFast Lys Kit (Roche) on MagNA Lyser (Roche). The DNA concentration was verified using Quant-iT PicoGreen dsDNA Kit (Thermo Fisher Scientific). Bacterial community composition was determined by paired-end sequencing on an Illumina MiSeq platform, targeting the V3–V4 hypervariable region of the 16S rRNA gene. Library preparation was carried out using the 341F (5′-CCTACGGGNGGCWGCAG-3′) – 805R (5′-GACTACHVGGGTATCTAATCC-3′) set of primers. On the same run a template-free sample was included as a negative sequencing control.

Sequencing data was deposited at metagenomic analysis server MG-RAST (Meyer et al., 2008) and is accessible at https://www.mg-rast.org/linkin.cgi?project=mgp85501.

### Analysis of Sequence Data

MiSeq output data was analyzed with statistical tools included in the mothur software (v.1.36.1) (Schloss et al., 2009), according to the MiSeq standard operating procedure (SOP) for Illumina paired end reads (Kozich et al., 2013). Sequences with any ambiguous base or more than eight homopolymers were removed from downstream analysis. The remaining sequences were aligned against the Silva reference alignment (Quast et al., 2013). Chimeras were searched with the UCHIME algorithm (Edgar et al., 2011) and removed. Classification of sequences was performed using the RDP training set (v.9) with an 80% confidence threshold. Sequences restricted to bacterial origin only were clustered into operational taxonomic units (OTUs) at a 3% dissimilarity level. To adjust for the influence of the number of OTUs in a sample on diversity and other statistical tests, any OTUs with relative abundance lower than 0.001% were removed from further analyses. Chao1 and Shannon indices were chosen to characterize alpha samples diversity. For beta diversity estimation weighted UniFrac measure was used (Lozupone et al., 2007). Samples hierarchical clustering was obtained with MEGA software (v.7.0.26) (Kumar et al., 2016) using Bray–Curtis distances as input. OTUs that differed between treatments were selected with respect to linear discriminant analysis (LDA) effect size (LEfSe) method (Segata et al., 2011).

## Results

### *C. difficile* Can Grow in the Presence of Dysbiotic and Healthy Fecal Microbiota *in Vitro*

Two types of fecal microbiota were tested. As explained previously one pooled sample was collected from healthy individuals while the other originated from samples subjected to *C. difficile* testing and were having negative result. Microbiota in such samples was previously and in this study shown to differ from microbiota in healthy samples (see below). Because the differences include characteristics associated with dysbalanced microbiota such as lower diversity and higher proportion of *Proteobacteria*, we are referring to this sample as to dysbiotic.

**FIGURE 1:**
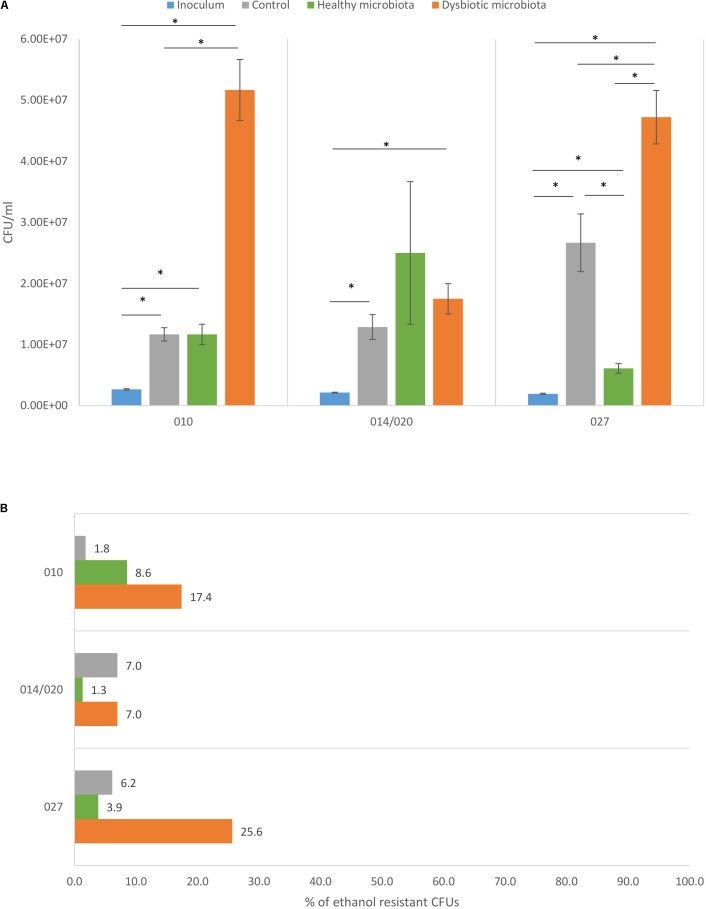
**(A)** Total viable cell count with corresponding standard deviation for *C. difficile* ribotypes 010, 014/020, and 027 strains in the inoculum (blue), in the control (gray), in co-culture with healthy fecal microbiota (green) and in co-culture with dysbiotic fecal microbiota (orange). ^∗^Statistically significant difference. **(B)** Percentage of *C. difficile* spores detected as ethanol resistant CFUs in proportion to total CFUs for ribotypes 010, 014/020, and 027 strains in the control (gray), in combination with healthy microbiota (green) and in combination with dysbiotic microbiota (orange).

Each tested *C. difficile* strain showed specific pattern of growth in the presence of healthy or dysbiotic microbiota. Strains of ribotypes 010 and 027 grew best in the presence of dysbiotic microbiota, while ribotype 014/020 strain growth was similar in both microbiota types (**Figure 1A**).

In both microbiota types an increase in *C. difficile* total cell count was observed compared to original inoculum, indicating that all three strains were able to proliferate in the presence of disturbed and undisturbed microbiota. Difference between growth in control (growth medium only) and in combination with microbiota (any type) was significant only for PCR ribotype 027 strain (**Figure 1A**).

### *C. difficile* Sporulation Is Better in the Presence of Dysbiotic Fecal Microbiota

The *C. difficile* sporulation frequency in co-cultures with fecal microbiota varied between 1.3 and 25.6% (**Figure 1B**). All three strains formed higher percentage of spores in co-culture with dysbiotic microbiota (7.0–25.6%), while in the co-culture with healthy microbiota spore percentage was lower (1.3–8.6%). Statistically significantly higher amount of spores in dysbiotic microbiota was observed only for ribotype 010 and 027 strains (one-way ANOVA: *p* < 0.05). Strains of ribotypes 014/020 and 027 displayed similar pattern of sporulation as for both the percentage of spores was the lowest in combination with healthy microbiota. For ribotype 010 strain the lowest sporulation was observed in control samples without fecal microbiota (**Figure 1B**).

### Bacterial Richness and Diversity of Fecal Microbiota Decrease in the Presence of *C. difficile*

Sequencing yielded a total of 1,480,790 sequences of bacterial origin, with an average depth of 50,385 sequences per sample. Sequences were classified into 365 OTUs with relative abundance higher than 0.001%.

**FIGURE 2:**
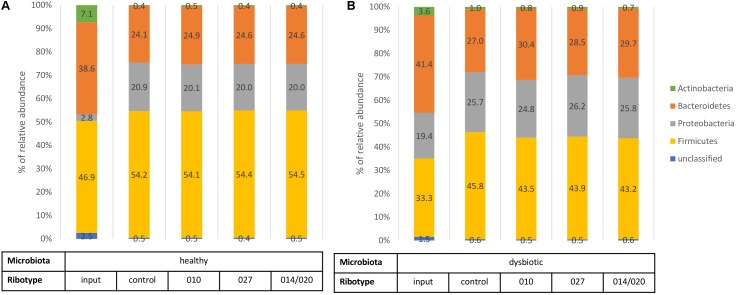
Bacterial phylum level assignments of average relative abundances for fecal input samples (before any cultivation), control samples (fecal microbiota only), and samples of fecal microbiota in combination with *C. difficile* ribotypes 010, 027, and 014/020 strains. Chart **(A)** represents samples of healthy fecal microbiota and chart **(B)** represents samples of dysbiotic fecal microbiota.

Input sample of healthy microbiota initially contained lower levels of *Proteobacteria* and higher proportions of *Actinobacteria* and *Firmicutes* than input sample of dysbiotic microbiota (**Figure 2**). After 3 days of *in vitro* incubation the control samples of both types of microbiota displayed similar composition at the phylum level (**Figure 2**). Nevertheless, statistical analysis revealed statistically higher bacterial richness and diversity in samples of healthy microbiota than in samples of dysbiotic microbiota (*p* < 0.05; Supplementary Table S1).

**FIGURE 3:**
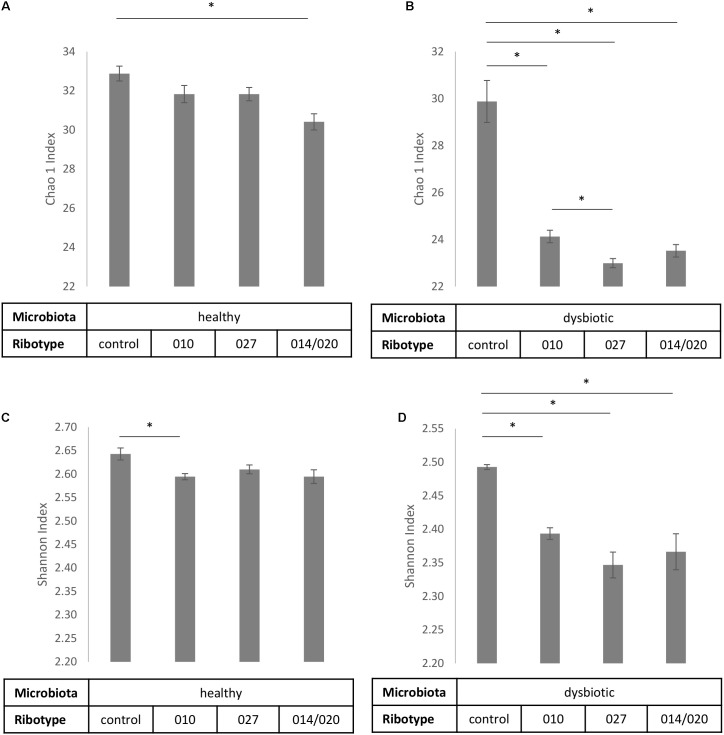
Alpha diversity estimates Chao1 **(A,B)** and Shannon index **(C,D)** with corresponding standard deviations for control samples of fecal microbiota and samples of fecal microbiota in combination with *C. difficile* ribotypes 010, 027, and 014/020 strains. Charts **(A,C)** represent samples of healthy fecal microbiota and charts **(B,D)** represent samples of dysbiotic fecal microbiota. ^∗^Statistically significant difference.

Also in comparison of control samples and co-cultures microbiota/*C. difficile* no statistically significant differences (*p* > 0.05) were detected at the phylum level (**Figure 2**), but significant changes were observed in bacterial richness and diversity (**Figure 3**) and on lower taxonomic levels (**Table 1**).

**Table 1:** Comparison of significantly increased OTUs in control samples (healthy or dysbiotic microbiota only) and in samples of co-cultures (healthy microbiota/*C. difficile* or dysbiotic microbiota/*C. difficile*).

Microbiota type +*C. difficile* ribotype	Increased in co-culture	Increased in control (microbiota only)
Healthy + 010	*Enterococcus* (Otu00004)	*Clostridium_XlVb* (Otu00043)
	*Barnesiella* (Otu00024)	*Coprococcus* (Otu00028)
	*Anaerostipes* (Otu00029)	*Morganella* (Otu00107)
	Uncl. from *Lachnospiraceae* (Otu00118)	*Coprococcus* (Otu00011)
	*Clostridium_XlVa* (Otu00032)	Uncl. from *Lachnospiraceae* (Otu00015)

Healthy + 014/020	*Enterococcus* (Otu00004)	*Clostridium_XlVb* (Otu00043)
	Uncl. from *Acidaminococcaceae* (Otu00068)	*Coprococcus* (Otu00011)
	*Barnesiella* (Otu00024)	*Coprococcus* (Otu00028)
	Uncl. from *Lachnospiraceae* (Otu00118)	*Morganella* (Otu00107)
	*Butyricicoccus* (Otu00051)	*Peptostreptococcus* (Otu00035)

Healthy + 027	*Enterococcus* (Otu00004)	*Clostridium_XlVb* (Otu00043)
	*Barnesiella* (Otu00024)	*Morganella* (Otu00107)
	*Clostridium_XlVa* (Otu00032)	*Coprococcus* (Otu00028)
	*Bacteroides* (Otu00048)	*Sutterella* (Otu00014)
		*Peptostreptococcus* (Otu00035)

Dysbiotic + 010	*Sutterella* (Otu00014)	*Dorea* (Otu00017)
	*Clostridium_sensu_stricto* (Otu00006)	*Escherichia_Shigella* (Otu00001)
	*Bacteroides* (Otu00002)	*Clostridium_XlVa* (Otu00032)
	*Veillonella* (Otu00003)	Uncl. from *Lachnospiraceae* (Otu00015)
	*Streptococcus* (Otu00012)	*Oscillibacter* (Otu00081)

Dysbiotic + 014/020	*Bacteroides* (Otu00002)	*Oscillibacter* (Otu00081)
	*Sutterella* (Otu00014)	*Clostridium_XlVa* (Otu00032)
	*Veillonella* (Otu00003)	Uncl. from *Ruminococcaceae* (Otu00030)
	*Bacteroides* (Otu00007)	Uncl. from *Eubacteriaceae* (Otu00094)
	*Parabacteroides* (Otu00021)	*Flavonifractor* (Otu00026)

Dysbiotic + 027	*Veillonella* (Otu00003)	*Dorea* (Otu00017)
	*Sutterella* (Otu00014)	*Clostridium_XlVa* (Otu00032)
	*Streptococcus* (Otu00012)	*Oscillibacter* (Otu00081)
	*Clostridium_sensu_stricto* (Otu00006)	Uncl. from *Lachnospiraceae* (Otu00015)
	*Phascolarctobacterium* (Otu00023)	*Flavonifractor* (Otu00026)


*Presented OTUs were identified by the LEfSe test (mothur software), which uses linear discriminant analysis (LDA) to find OTUs that significantly differ in abundance between cultures of microbiota only (control) and co-cultures of microbiota and *C. difficile*. For each comparison only top five OTUs with highest LDA scores are presented. For additional information, see Supplementary Tables S2, S3. Uncl., unclassified.*

To estimate the microbial richness we used the Chao1 index. According to control samples with healthy microbiota alone significantly lower bacterial richness was observed only if healthy microbiota was co-cultured with ribotype 014/020 strain (**Figure 3A**), whereas bacterial richness of dysbiotic microbiota was lowered in the presence of all three strains (**Figure 3B**). In addition, significant differences in bacterial richness were observed between co-culture of dysbiotic microbiota with ribotype 027 strain and co-culture of dysbiotic microbiota with ribotype 010 strain (**Figure 3B**).

Shannon index, predicting the microbial diversity, was significantly higher in control samples than in co-cultures microbiota/*C. difficile* (**Figures 3C,D**). For dysbiotic microbiota that trend was noticeable for all three strains, whereas bacterial diversity of healthy microbiota significantly decreased only in co-culture with ribotype 010 strain.

### Dysbiotic Microbiota Is More Affected by *C. difficile* Than Healthy Microbiota

**FIGURE 4:**
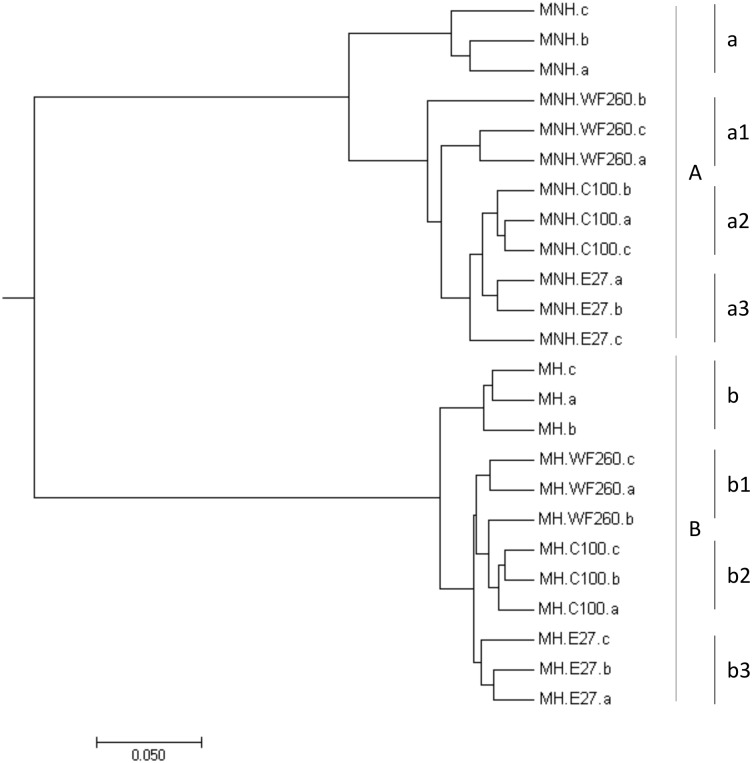
Hierarchical clustering of samples of dysbiotic fecal microbiota (MNH; segment A) and samples of healthy fecal microbiota (MH; segment B) without and with the addition of *C. difficile* ribotype 010 strain (C100), ribotype 027 strain (E27), and ribotype 014/020 strain (WF260), obtained by MEGA software (v.7.0.26). Statistically significant differences were observed between control samples (dysbiotic or healthy fecal microbiota only; segments a and b) and samples of fecal microbiota (dysbiotic or healthy) with added *C. difficile* ribotypes 010 (segments a2 and b2), 014/020 (segments a1 and b1), and 027 (segments a3 and b3) strains.

Bacterial community patterns of control and co-culture samples congregated in two major groups by hierarchical clustering; i.e., samples of dysbiotic microbiota (**Figure 4**, segment A) and samples of healthy microbiota (**Figure 4**, segment B). The deepest branching in both groups clearly divided control samples of microbiota only from co-cultures microbiota/*C. difficile* (**Figure 4**). According to weighted UniFrac test statistically significant differences in OTU composition were observed between control samples (dysbiotic or healthy microbiota only; **Figure 4**, segments a and b) and samples of microbiota (dysbiotic or healthy) in co-culture with all three *C. difficile* ribotypes (**Figure 4**, segments a1–3 and b1–3).

To identify significantly changed OTUs we have used the LEfSe test. Altogether, 62 OTUs were affected in co-cultures of dysbiotic microbiota and 45 OTUs in co-cultures of healthy microbiota (Supplementary Tables S2, S3). The smallest number of OTUs was affected in co-culture of healthy microbiota and *C. difficile* ribotype 027 strain (Supplementary Table S3). In general, the majority of significantly changed OTUs in both types of microbiota belonged to the phylum *Firmicutes*, with *Lachnospiraceae* and *Ruminococcaceae* origin. OTUs from both families were mostly associated with control samples of microbiota only (**Table 1** and Supplementary Tables S2, S3). For *Clostridium_XIVa*, member of *Lachnospiraceae* family, an increase was observed in control samples of dysbiotic microbiota only as well as in co-cultures of healthy microbiota and *C. difficile* (**Table 1** and Supplementary Tables S2, S3). Members of *Bacteroidetes* (particularly *Bacteroides*), *Firmicutes* (e.g., *Veillonella*) and *Proteobacteria* (i.e., *Sutterella*) were mostly associated with co-cultures of dysbiotic microbiota/*C. difficile*, whereas in co-cultures of healthy microbiota/*C. difficile* taxa from *Bacteroidetes* (i.e., *Barnesiella*) and *Firmicutes* (e.g., *Enterococcus*) were enriched (**Table 1**).

## Discussion

Widespread use of antibiotics in hospitalized as well as in non-hospitalized patients leads to the disturbance of normal gut microbiota and allows *C. difficile* to expand in the gut. However, CDI might occur also in the individuals with no previous exposure to antibiotics or other apparent reason for microbiota disruption (Bauer et al., 2008; Wilcox et al., 2008).

To partially address the issue of *C. difficile* proliferation in dysbiotic and non-dysbiotic gut microenvironment, we explored *C. difficile* growth in the presence of two different types of microbiota. As previously *C. difficile* conditioned medium was shown to cause changes in gut microbiota *in vitro* (Horvat et al., 2017), we have studied if comparable changes are observed also with *C. difficile* vegetative cells.

All tested *C. difficile* strains successfully proliferated in our *in vitro* model with both types of fecal microbiota. That is in contrast to results obtained with some other *in vitro* gut models, where the inability of *C. difficile* to proliferate in the absence of antibiotic disturbance of microbiota was demonstrated (Borriello and Barclay, 1986; Wilson and Freter, 1986; Drummond et al., 2003; Freeman et al., 2003). Yet, the capability of *C. difficile* to colonize gut microenvironments even in the presence of complex microbial communities was demonstrated for ribotype 027 strains (Robinson et al., 2014). Therefore, it seems likely that ability of *C. difficile* to compete with complex non-dysbiotic microbial communities is strain dependent.

With the exception of ribotype 014/020 strain, *C. difficile* proliferated to a significantly greater extent in the presence of dysbiotic microbiota than in the presence of healthy microbiota (**Figure 1A**). In addition, greater proportion of OTUs was affected in co-cultures dysbiotic microbiota/*C. difficile* than in co-cultures healthy microbiota/*C. difficile* (Supplementary Tables S2, S3). Interestingly, the presence of ribotype 027 in healthy microbiota affected only small proportion of OTUs (Supplementary Table S3). That is in contrast with several previous studies on symptomatic humans or animals where ribotype 027 correlated with more disturbed microbiota (Lawley et al., 2012; Rea et al., 2012; Skraban et al., 2013).

Considering sporulation, higher proportion of *C. difficile* spores was observed in co-cultures with dysbiotic microbiota. That trend was apparent for all three strains (**Figure 1B**). This indicates, that conditions in dysbiotic microbiota support formation of greater amount of spores, which are important for further *C. difficile* transmission and spread. Additionally, an increased spore production appears to be relevant in severe CDI cases (Carlson et al., 2013).

The explanation of mechanism underlying the observed increased sporulation could be only speculative, as sporulation signals for *C. difficile* are not known (Zhu et al., 2018) in contrast to well understood germination signals (Theriot and Young, 2014). Nutrient starvation, quorum sensing and other environmental stimuli are most likely involved in sporulation signaling through several regulatory networks (Zhu et al., 2018). All of these three potential factors could be also involved in changes of microbiota dependent sporulation frequency.

Presence of *C. difficile* in co-cultures significantly decreased microbial richness and diversity in both types of fecal microbiota, but dysbiotic microbiota seemed to be more responsive and healthy microbiota more robust (**Figure 3**). Diversity was decreased in the presence of all three tested strains. The association of *C. difficile* colonization and/or infection with a decrease in gut microbiota diversity is well documented, but was so far only viewed as a predisposing condition (reviewed in Vincent and Manges, 2015) and not also as a consequence of *C. difficile* presence as suggested here and elsewhere (Horvat et al., 2017).

In general, potentially beneficial autochthonous bacteria, particularly butyrate-producing bacteria (*Lachnospiraceae* and *Ruminococcaceae*) were present in larger proportions in microbiota cultivated without *C. difficile*. These bacteria are usually found in healthy subjects and are thought to provide colonization resistance against CDI (Ross et al., 2016).

The presence of *C. difficile* in any type of microbiota resulted in increased proportions of opportunistic pathogens (i.e., *Sutterella* in co-culture with dysbiotic microbiota and *Enterococcus* in co-culture with healthy microbiota). Previous studies already reported about an increased proportion of *Sutterella* in *C. difficile* positive subjects (Perez-Cobas et al., 2014; Milani et al., 2016). Also, the presence of *Sutterella* was linked to several other human diseases, where gut microbiota seems to play a crucial role in their development (e.g., metabolic syndrome and autism) (Wang et al., 2013; Lim et al., 2017). An association between *C. difficile* colonization/infection and enterococci or vancomycin-resistant enterococci (VRE) is also well documented (Ozaki et al., 2004; Choi et al., 2011; Fujitani et al., 2011; Stripling et al., 2015; McKinley et al., 2016). Moreover, our previous study demonstrated undisturbed growth of representative strain of *Enterococcus* in co-culture with *C. difficile* on agar plates (Horvat et al., 2017). *Veillonella* was another genus that increased in the presence of *C. difficile* in dysbiotic microbiota. That is in agreement with two other studies (Antharam et al., 2016; Khanna et al., 2016) and in contrast to our previous study, where reduced proportions were found in *C. difficile* conditioned medium (Horvat et al., 2017). Decreased levels of *Veillonella* were also found in oncological patients colonized with *C. difficile* (Zwielehner et al., 2011).

Our results further support the observation that *C. difficile* could have an impact on gut microbiota and that the impact is strain dependent. Dysbiotic microbiota is *in vitro* more susceptible for disturbances caused by co-culture with *C. difficile* and is also associated with better sporulation frequency of *C. difficile*. Better competitive ability, inhibition of certain bacterial groups associated with colonization resistance and better sporulation in the presence of dysbiotic microbiota possibly contribute to endemic spread of some strains.

## Author Contributions

SH performed the experiments and data analysis. MR contributed to the data analysis. Both authors designed the study and contributed to the text preparation and reviewed the manuscript.

## Conflict of Interest Statement

The authors declare that the research was conducted in the absence of any commercial or financial relationships that could be construed as a potential conflict of interest.
