# Supplementary material for: Interactions Between Clostridioides difficile and Fecal Microbiota in in Vitro Batch Model: Growth, Sporulation, and Microbiota Changes
Source: Front Microbiol. 2018 Jul 24;9:1633. doi: 10.3389/fmicb.2018.01633 (PMC6066498; doi:10.3389/fmicb.2018.01633)
Supplement: Supplementary file 1 [file Table_1.DOCX]

***Supplementary Material***

**Interactions between *Clostridium difficile* and fecal microbiota in *in vitro* batch model: growth, sporulation and microbiota changes**

**Sabina Horvat and Maja Rupnik^*^**

^*^**Correspondence**: Corresponding Author: [maja.rupnik@nlzoh.si](mailto:maja.rupnik@nlzoh.si)

**Table S1:** Comparison of alpha diversity estimates Chao1 and Shannon index between control samples of fecal microbiota (healthy and dysbiotic), with corresponding p-value.

|  | healthy | dysbiotic | p-value |
| --- | --- | --- | --- |
| Chao 1* | 33 (22, 77) | 30 (20, 72) | 0.028 |
| Shannon** | 2.64 ± 0.01 | 2.49 ± 0.00 | 0.002 |

*The values in parentheses are the 95% confidence intervals. **Mean value with corresponding standard deviation.

**Table S2**: OTUs that significantly differ in abundance between cultures of dysbiotic microbiota only (control) and co-cultures of dysbiotic fecal microbiota and *C. difficile* ribotypes 010, 014/020 and 027 strains, identified by the LEfSe test (mothur software). OTUs with negative LDA scores (red) are enriched in samples of dysbiotic fecal microbiota with added *C. difficile*, while OTUs with positive LDA scores (blue) are enriched in samples of dysbiotic fecal microbiota only. OTUs are listed according to LDA score in a descending manner.

| comparison | dysbiotic vs dysbiotic + 010 | dysbiotic vs  dysbiotic + 014/020 | dysbiotic vs dysbiotic + 027 |  |  |  |
| --- | --- | --- | --- | --- | --- | --- |
| WUniFrac | < 0.001 | < 0.001 | < 0.001 | Phylum | Family | Genus |
| Otu00032 | 3.88 | 3.78 | 3.95 | Firmicutes | Lachnospiraceae | Clostridium_XlVa |
| Otu00081 | 3.79 | 3.79 | 3.79 | Firmicutes | Ruminococcaceae | Oscillibacter |
| Otu00094 | 3.64 | 3.66 | 3.64 | Firmicutes | Eubacteriaceae | uncl. from Eubacteriaceae |
| Otu00026 | 3.59 | 3.63 | 3.65 | Firmicutes | Ruminococcaceae | Flavonifractor |
| Otu00008 | 3.63 | 3.57 | 3.60 | Proteobacteria | Sutterellaceae | Sutterella |
| Otu00030 | 3.52 | 3.70 | 3.51 | Firmicutes | Ruminococcaceae | uncl. from Ruminococcaceae |
| Otu00025 | 3.41 | 3.52 | 3.45 | Firmicutes | Lachnospiraceae | Dorea |
| Otu00045 | 3.26 | 3.31 | 3.39 | Firmicutes | Lachnospiraceae | Clostridium_XlVa |
| Otu00057 | 3.33 | 3.28 | 3.28 | Firmicutes | Ruminococcaceae | uncl. from Ruminococcaceae |
| Otu00046 | 3.11 | 3.23 | 3.08 | Bacteroidetes | Rikenellaceae | Alistipes |
| Otu00071 | 2.92 | 2.92 | 3.11 | Proteobacteria | Sutterellaceae | Parasutterella |
| Otu00060 | 2.88 | 2.96 | 2.91 | Bacteroidetes | Rikenellaceae | Alistipes |
| Otu00106 | 2.92 | 2.91 | 2.92 | Firmicutes | Ruminococcaceae | Oscillibacter |
| Otu00103 | 2.95 | 2.93 | 2.77 | Firmicutes | Acidaminococcaceae | Acidaminococcus |
| Otu00063 | 2.89 | 2.95 | 2.79 | Firmicutes | Lachnospiraceae | uncl. from Lachnospiraceae |
| Otu00017 | 4.28 | 0.00 | 4.30 | Firmicutes | Lachnospiraceae | Dorea |
| Otu00146 | 2.77 | 2.68 | 2.84 | Firmicutes | Ruminococcaceae | uncl. from Ruminococcaceae |
| Otu00028 | 2.53 | 2.83 | 2.70 | Firmicutes | Lachnospiraceae | Coprococcus |
| Otu00015 | 3.82 | 0.00 | 3.72 | Firmicutes | Lachnospiraceae | uncl. from Lachnospiraceae |
| Otu00054 | 2.39 | 2.37 | 2.48 | Firmicutes | Lachnospiraceae | Blautia |
| Otu00201 | 2.39 | 2.31 | 2.39 | Proteobacteria | Sutterellaceae | Parasutterella |
| Otu00127 | 2.38 | 2.26 | 2.31 | Firmicutes | Lachnospiraceae | uncl. from Lachnospiraceae |
| Otu00166 | 2.15 | 2.07 | 2.50 | Firmicutes | Erysipelotrichaceae | Holdemania |
| Otu00202 | 2.18 | 2.24 | 2.29 | Firmicutes | Lachnospiraceae | uncl. from Lachnospiraceae |
| Otu00059 | 3.05 | 3.17 | 0.00 | Actinobacteria | Coriobacteriaceae | uncl. from Coriobacteriaceae |
| Otu00178 | 0.00 | 2.56 | 2.62 | Firmicutes | Lachnospiraceae | uncl. from Lachnospiraceae |
| Otu00055 | 2.36 | 2.44 | 0.00 | Firmicutes | Lachnospiraceae | Roseburia |
| Otu00001 | 3.94 | 0.00 | 0.00 | Proteobacteria | Enterobacteriaceae | Escherichia_Shigella |
| Otu00023 | 3.08 | 3.38 | -2.73 | Firmicutes | Acidaminococcaceae | Phascolarctobacterium |
| Otu00004 | 0.00 | 0.00 | 3.16 | Firmicutes | Enterococcaceae | Enterococcus |
| Otu00034 | 0.00 | 0.00 | 2.99 | Firmicutes | Erysipelotrichaceae | Clostridium_XVIII |
| Otu00091 | 0.00 | 2.48 | 0.00 | Firmicutes | Clostridiaceae | Clostridium_sensu_stricto |
| Otu00005 | 0.00 | 0.00 | 2.46 | Firmicutes | Lachnospiraceae | Clostridium_XlVa |
| Otu00247 | 0.00 | 0.00 | 2.29 | uncl. | uncl. | uncl. |
| Otu00022 | 0.00 | 2.28 | 0.00 | Bacteroidetes | Bacteroidaceae | Bacteroides |
| Otu00167 | 2.24 | 0.00 | 0.00 | Firmicutes | Ruminococcaceae | uncl. from Ruminococcaceae |
| Otu00177 | 0.00 | 2.14 | 0.00 | Firmicutes | Lachnospiraceae | Clostridium_XlVa |
| Otu00111 | 0.00 | 2.11 | 0.00 | Proteobacteria | Enterobacteriaceae | Enterobacter |
| Otu00069 | 0.00 | 2.06 | 0.00 | Proteobacteria | Enterobacteriaceae | uncl. from Enterobacteriaceae |
| Otu00191 | 2.02 | 0.00 | 0.00 | Firmicutes | Ruminococcaceae | Flavonifractor |
| Otu00150 | -2.08 | 0.00 | 0.00 | Firmicutes | Erysipelotrichaceae | Erysipelotrichaceae_incertae_sedis |
| Otu00089 | -2.13 | 0.00 | 0.00 | Firmicutes | Ruminococcaceae | uncl. from Ruminococcaceae |
| Otu00153 | 0.00 | 0.00 | -2.17 | Firmicutes | Erysipelotrichaceae | Erysipelotrichaceae_incertae_sedis |
| Otu00011 | 0.00 | -2.19 | 0.00 | Firmicutes | Lachnospiraceae | Coprococcus |
| Otu00042 | -2.40 | 0.00 | 0.00 | Bacteroidetes | Bacteroidaceae | Bacteroides |
| Otu00108 | 0.00 | 0.00 | -2.49 | Firmicutes | Streptococcaceae | Streptococcus |
| Otu00125 | 0.00 | -2.49 | 0.00 | Firmicutes | Lactobacillaceae | Lactobacillus |
| Otu00319 | -2.72 | 0.00 | 0.00 | Bacteroidetes | Bacteroidaceae | Bacteroides |
| Otu00020 | -2.72 | 0.00 | 0.00 | Bacteroidetes | Porphyromonadaceae | Parabacteroides |
| Otu00024 | -3.09 | 0.00 | 0.00 | Bacteroidetes | Porphyromonadaceae | Barnesiella |
| Otu00157 | -2.06 | 0.00 | -2.40 | Bacteroidetes | Porphyromonadaceae | Butyricimonas |
| Otu00062 | -2.71 | -2.87 | 0.00 | Firmicutes | Ruminococcaceae | uncl. from Ruminococcaceae |
| Otu00037 | -2.94 | -2.89 | 0.00 | Firmicutes | Lachnospiraceae | Blautia |
| Otu00021 | -3.28 | -3.19 | 0.00 | Bacteroidetes | Porphyromonadaceae | Parabacteroides |
| Otu00119 | -2.56 | -2.33 | -2.36 | Bacteroidetes | Porphyromonadaceae | uncl. from Porphyromonadaceae |
| Otu00007 | -3.78 | -3.56 | 0.00 | Bacteroidetes | Bacteroidaceae | Bacteroides |
| Otu00074 | -2.35 | -2.65 | -2.52 | Bacteroidetes | Bacteroidaceae | Bacteroides |
| Otu00006 | -3.94 | 0.00 | -3.67 | Firmicutes | Clostridiaceae | Clostridium_sensu_stricto |
| Otu00002 | -3.85 | -3.99 | 0.00 | Bacteroidetes | Bacteroidaceae | Bacteroides |
| Otu00012 | -3.80 | -3.11 | -3.77 | Firmicutes | Streptococcaceae | Streptococcus |
| Otu00003 | -3.85 | -3.62 | -4.01 | Firmicutes | Veillonellaceae | Veillonella |
| Otu00014 | -3.96 | -3.95 | -3.85 | Proteobacteria | Sutterellaceae | Sutterella |

uncl.=unclassified; WUniFrac=weighted UniFrac

**Table S3**: OTUs that significantly differ in abundance between cultures of healthy microbiota only (control) and co-cultures of healthy fecal microbiota and *C. difficile* ribotypes 010, 014/020 and 027 strains, identified by the LEfSe test (mothur software). OTUs with negative LDA scores (red) are enriched in samples of healthy fecal microbiota with added *C. difficile*, while OTUs with positive LDA scores (blue) are enriched in samples of healthy fecal microbiota only. OTUs are listed according to LDA score in a descending manner.

| comparison | healthy vs healthy + 010 | healthy vs  healthy + 014/020 | healthy vs  healthy + 027 |  |  |  |
| --- | --- | --- | --- | --- | --- | --- |
| WUniFrac | < 0.001 | < 0.001 | < 0.001 | Phylum | Family | Genus |
| Otu00043 | 4.05 | 4.04 | 4.05 | Firmicutes | Lachnospiraceae | Clostridium_XlVb |
| Otu00028 | 3.41 | 3.47 | 3.27 | Firmicutes | Lachnospiraceae | Coprococcus |
| Otu00107 | 3.37 | 3.37 | 3.28 | Proteobacteria | Enterobacteriaceae | Morganella |
| Otu00072 | 3.09 | 3.06 | 2.81 | Proteobacteria | Desulfovibrionaceae | Desulfovibrio |
| Otu00114 | 2.97 | 2.98 | 2.82 | Firmicutes | Lachnospiraceae | uncl. from Lachnospiraceae |
| Otu00046 | 2.55 | 2.63 | 2.60 | Bacteroidetes | Rikenellaceae | Alistipes |
| Otu00078 | 2.43 | 2.46 | 2.46 | Firmicutes | Erysipelotrichaceae | Clostridium_XVIII |
| Otu00077 | 2.37 | 2.55 | 2.39 | Firmicutes | Clostridiaceae | Clostridium_sensu_stricto |
| Otu00011 | 3.29 | 3.47 | 0.00 | Firmicutes | Lachnospiraceae | Coprococcus |
| Otu00015 | 3.28 | 3.16 | 0.00 | Firmicutes | Lachnospiraceae | uncl. from Lachnospiraceae |
| Otu00035 | 0.00 | 3.26 | 2.88 | Firmicutes | Peptostreptococcaceae | Peptostreptococcus |
| Otu00026 | 3.03 | 2.96 | 0.00 | Firmicutes | Ruminococcaceae | Flavonifractor |
| Otu00063 | 2.72 | 2.82 | 0.00 | Firmicutes | Lachnospiraceae | uncl. from Lachnospiraceae |
| Otu00030 | 2.77 | 2.75 | 0.00 | Firmicutes | Ruminococcaceae | uncl. from Ruminococcaceae |
| Otu00054 | 2.67 | 2.75 | 0.00 | Firmicutes | Lachnospiraceae | Blautia |
| Otu00087 | 2.67 | 2.71 | 0.00 | Firmicutes | Ruminococcaceae | Flavonifractor |
| Otu00034 | 2.65 | 2.73 | 0.00 | Firmicutes | Erysipelotrichaceae | Clostridium_XVIII |
| Otu00057 | 2.53 | 0.00 | 2.81 | Firmicutes | Ruminococcaceae | uncl. from Ruminococcaceae |
| Otu00137 | 2.59 | 2.69 | 0.00 | Firmicutes | Lachnospiraceae | Clostridium_XlVb |
| Otu00095 | 2.73 | 2.44 | 0.00 | Firmicutes | Lachnospiraceae | uncl. from Lachnospiraceae |
| Otu00012 | 2.46 | 2.54 | 0.00 | Firmicutes | Streptococcaceae | Streptococcus |
| Otu00133 | 2.27 | 2.31 | 0.00 | Firmicutes | Ruminococcaceae | uncl. from Ruminococcaceae |
| Otu00167 | 2.21 | 2.21 | 0.00 | Firmicutes | Ruminococcaceae | uncl. from Ruminococcaceae |
| Otu00126 | 2.37 | 2.01 | 0.00 | Firmicutes | Ruminococcaceae | uncl. from Ruminococcaceae |
| Otu00060 | 2.18 | 2.18 | 0.00 | Bacteroidetes | Rikenellaceae | Alistipes |
| Otu00019 | 0.00 | 3.18 | 0.00 | Firmicutes | Peptostreptococcaceae | Clostridium_XI |
| Otu00014 | 0.00 | 0.00 | 3.18 | Proteobacteria | Sutterellaceae | Sutterella |
| Otu00009 | 0.00 | 3.16 | 0.00 | Bacteroidetes | Bacteroidaceae | Bacteroides |
| Otu00065 | 0.00 | 2.77 | 0.00 | Firmicutes | Ruminococcaceae | Oscillibacter |
| Otu00025 | 2.66 | 0.00 | 0.00 | Firmicutes | Lachnospiraceae | Dorea |
| Otu00092 | 0.00 | 2.40 | 0.00 | Firmicutes | Lachnospiraceae | uncl. from Lachnospiraceae |
| Otu00081 | 2.20 | 0.00 | 0.00 | Firmicutes | Ruminococcaceae | Oscillibacter |
| Otu00082 | 0.00 | 2.13 | 0.00 | Bacteroidetes | Prevotellaceae | Prevotella |
| Otu00048 | 0.00 | 0.00 | -2.35 | Bacteroidetes | Bacteroidaceae | Bacteroides |
| Otu00016 | 0.00 | -2.76 | 0.00 | Bacteroidetes | Bacteroidaceae | Bacteroides |
| Otu00029 | -3.20 | 0.00 | 0.00 | Firmicutes | Lachnospiraceae | Anaerostipes |
| Otu00068 | 0.00 | -3.63 | 0.00 | Firmicutes | Acidaminococcaceae | uncl. from Acidaminococcaceae |
| Otu00036 | -2.19 | -2.18 | 0.00 | Firmicutes | Lachnospiraceae | Blautia |
| Otu00037 | -2.59 | -2.46 | 0.00 | Firmicutes | Lachnospiraceae | Blautia |
| Otu00053 | -2.71 | -2.61 | 0.00 | Firmicutes | Lachnospiraceae | Lachnospiracea_incertae_sedis |
| Otu00051 | -2.54 | -2.92 | 0.00 | Firmicutes | Ruminococcaceae | Butyricicoccus |
| Otu00118 | -2.99 | -2.96 | 0.00 | Firmicutes | Lachnospiraceae | uncl. from Lachnospiraceae |
| Otu00032 | -2.75 | -2.77 | -2.57 | Firmicutes | Lachnospiraceae | Clostridium_XlVa |
| Otu00024 | -3.58 | -3.41 | -3.33 | Bacteroidetes | Porphyromonadaceae | Barnesiella |
| Otu00004 | -3.99 | -3.97 | -3.68 | Firmicutes | Enterococcaceae | Enterococcus |

uncl.=unclassified; WUniFrac=weighted UniFrac
